# Myricetin Modulates Quorum Sensing-Associated Phenotypes, Virulence, and Biofilm Formation in Pathogenic Bacteria: In Vitro and In Silico Studies

**DOI:** 10.3390/pharmaceutics18101280

**Published:** 2026-10-09

**Authors:** Abdulrahman S. Bazaid, Nizar H. Saeedi, Mohammed M. Jalal, Almohanad A. Alkayyal, Mitesh Patel, Abdu Aldarhami

**Affiliations:** 1 Department of Medical Laboratory Science, College of Applied Medical Sciences, University of Ha’il, Hail 55476, Saudi Arabia; ar.bazaid@uoh.edu.sa; 2 Medical and Diagnostic Research Center, University of Ha’il, Hail 55473, Saudi Arabia; 3 Department of Medical Laboratory Technology, Faculty of Applied Medical Sciences, University of Tabuk, Tabuk 71491, Saudi Arabia; nsaeedi@ut.edu.sa (N.H.S.); mjalal@ut.edu.sa (M.M.J.); aalkayyal@ut.edu.sa (A.A.A.); 4 Department of Bioinformatics, Faculty of Engineering and Technology, Marwadi University, Rajkot 360003, Gujarat, India; patelmeet15@gmail.com; 5 Department of Microbiology and Parasitology, Faculty of Medicine, Umm Al-Qura University, Al-Qunfudah 21961, Saudi Arabia

**Keywords:** myricetin, QS-associated phenotypes, QS modulation, antimicrobial resistance (AMR), biofilm formation inhibition, anti-virulence strategy

## Abstract

**Background/Objectives:** The rapid emergence of antimicrobial resistance (AMR) among pathogenic bacteria has necessitated the development of alternative therapeutic strategies that target bacterial virulence rather than cell viability. **Methods:** In this study, the flavonoid myricetin was systematically evaluated for its antibacterial, QS-associated phenotypic and biofilm formation-inhibitory activities against clinically relevant bacterial pathogens. **Results:** Myricetin exhibited moderate antibacterial activity and significantly reduced several QS-associated phenotypes. Violacein and prodigiosin production were reduced by 74.59% and 64.32%, respectively, consistent with QS-modulatory activity under the tested conditions. Furthermore, myricetin markedly attenuated key virulence factors in *Pseudomonas aeruginosa*, including pyocyanin (70.44%), pyoverdine (75.84%), and rhamnolipids (48.69%). In addition, concentration-dependent reductions in biofilm formation and extracellular polymeric substance (EPS) production was observed across all tested strains. Molecular docking predicted favourable interactions of myricetin with key QS regulatory proteins, particularly CviR and LasR, which was supported by molecular dynamics simulations. Notably, the LasR–myricetin complex showed greater computational stability among the evaluated complexes, supporting its potential role as a key interaction site. **Conclusions:** This study provides an integrated experimental and computational evaluation of myricetin-mediated modulation of QS-associated phenotypes across multiple bacterial systems, demonstrating reductions in virulence-factor production and biofilm formation under the tested conditions. Collectively, these findings position myricetin as a promising anti-virulence candidate, although further studies are required to validate its clinical applicability.

## 1. Introduction

The rapid emergence of antimicrobial resistance (AMR) poses a significant global health challenge, limiting the effectiveness of conventional antibiotic therapies [1]. Gram-negative pathogens, particularly Pseudomonas aeruginosa and Serratia marcescens, are frequently associated with persistent infections, hospital-acquired diseases, and treatment failures [2,3,4]. Their pathogenic success is largely attributed to their ability to regulate virulence and adapt to hostile environments.

A central mechanism underlying bacterial pathogenicity is quorum sensing (QS), a cell-density-dependent communication system that coordinates the expression of genes involved in virulence, biofilm formation, motility, and toxin production [5,6,7]. In *P. aeruginosa*, QS systems such as Las and Rhl regulate the production of multiple virulence factors, including pyocyanin, pyoverdine, and rhamnolipids [8,9]. In addition, QS plays a crucial role in biofilm development, where bacterial cells are embedded within an extracellular polymeric substance (EPS) matrix that enhances resistance to antibiotics and host immune responses [10,11].

Targeting QS has therefore emerged as a promising anti-virulence strategy, as it interferes with pathogenicity without directly affecting bacterial growth, potentially reducing selective pressure for resistance development [5,7]. Inhibition of QS-regulated pathways can attenuate virulence factor production, disrupt biofilm formation, and enhance the efficacy of conventional antimicrobial therapies [6,12]. Consequently, there is growing interest in identifying natural compounds capable of modulating QS systems.

Flavonoids, a diverse group of plant-derived polyphenolic compounds, have gained attention due to their antimicrobial, antioxidant, and anti-inflammatory activities [13,14]. Among them, myricetin, a hydroxylated flavonol widely distributed in fruits, vegetables, and medicinal plants, has demonstrated notable biological properties, including antimicrobial effects [15,16]. Although previous studies have reported antibacterial, biofilm formation-inhibitory and QS-associated effects of myricetin, including a recent study demonstrating inhibition of biofilm formation, QS-associated phenotypes and virulence-factor production in *P. aeruginosa* [16], the activity of myricetin across multiple bacterial species and QS-associated phenotypes remains comparatively less characterized. In addition, the integration of experimental evaluation across Gram-positive and Gram-negative bacteria with structure-based computational analysis of multiple QS- and virulence-associated protein targets has not been comprehensively investigated in a single study. Therefore, the present study was designed to extend the existing evidence by evaluating myricetin against multiple pathogenic bacterial species and by integrating phenotypic assays with molecular docking and molecular dynamics analyses to explore potential protein-level interactions.

A recent study by Al Hashedi et al. [16] reported that myricetin inhibits biofilm formation, QS-associated phenotypes and several virulence factors in *P. aeruginosa*, including pyocyanin, rhamnolipid and exopolysaccharide production, and further demonstrated changes in QS-related gene expression [16]. These findings establish that the anti-QS and biofilm formation-inhibitory potential of myricetin in *P. aeruginosa* has already been investigated. The present study therefore does not claim to establish these effects for the first time. Instead, its novelty lies in extending the experimental evaluation of myricetin to multiple bacterial species, including *S. aureus*, *E. coli*, *C. violaceum*, *P. aeruginosa*, *and S. marcescens*, and in integrating these phenotypic observations with computational investigation of multiple QS-, virulence and biofilm-associated protein targets. This broader multi-species and integrated experimental–computational framework provides additional evidence regarding the potential spectrum of myricetin-associated anti-virulence and biofilm-modulatory activity.

## 2. Materials and Methods

### 2.1. Myricetin Preparation

Myricetin (≥98% purity) was purchased from [Sigma-Aldrich, St. Louis, MO, USA]. A stock solution was prepared by dissolving myricetin in dimethyl sulfoxide (DMSO) to obtain a concentration of 10 mg/mL. The solution was vortexed to ensure complete dissolution and filtered using a 0.22 µm syringe filter for sterilization. Working concentrations were prepared by diluting the stock solution in appropriate culture media. Because the myricetin stock solution was prepared in 100% DMSO, the final DMSO concentration in each assay depended on the concentration of myricetin used. Therefore, concentration-matched DMSO vehicle controls were included for each corresponding myricetin treatment. The final DMSO concentrations corresponding to the 1/2, 1/4 and 1/8 MIC treatments were calculated based on the concentration of the myricetin stock solution and were used to prepare the respective vehicle controls.

### 2.2. Bacterial Strains

The antibacterial and anti-virulence activities of myricetin were evaluated against a panel of Gram-positive and Gram-negative bacterial strains, including *Staphylococcus aureus* MTCC-96, *Escherichia coli* MTCC-9537, *Chromobacterium violaceum* MTCC-2656, *Pseudomonas aeruginosa* MTCC-741, and *Serratia marcescens* MTCC-97. All strains were obtained from the Microbial Type Culture Collection (MTCC), and the complete accession numbers are provided to ensure unambiguous strain identification. *C. violaceum* was used as a QS reporter strain for assessment of violacein production, whereas the other strains were used to evaluate antibacterial, QS-associated, virulence-factor, biofilm formation, and/or EPS-related phenotypes as described below. Bacterial cultures were maintained on Mueller–Hinton agar (MHA) and subcultured prior to each experiment. A single colony was inoculated into fresh Mueller–Hinton broth (MHB) and incubated overnight at 37 °C. The bacterial suspension was adjusted to 0.5 McFarland (~1 × 10^8^ CFU/mL) using sterile saline.

### 2.3. Agar Well Diffusion Assay

The antibacterial activity of myricetin was assessed using the agar well diffusion method on MHA plates. Briefly, 100 µL of standardized bacterial suspension (0.5 McFarland) was uniformly spread onto MHA plates. Wells (6 mm diameter) were aseptically punched into the agar and filled with 50 µL of myricetin solution (1000 µg/mL). Plates were incubation at 37 °C for 24 h, after which zones of inhibition were measured in millimetres (mm). Streptomycin (1000 µg/mL) was used as positive control, while 10% DMSO, corresponding to the vehicle concentration present in the 1000 µg/mL myricetin treatment, was used as the concentration-matched vehicle control [17]. All experiments were performed in triplicate.

### 2.4. Minimum Inhibitory Concentration (MIC)

The MIC of myricetin was determined by the broth microdilution method according to CLSI guidelines [17]. Serial two-fold dilutions of myricetin (1000–1.95 µg/mL) were prepared in Mueller-Hinton broth (MHB) in 96-well microtiter plates. A bacterial suspension was prepared from a fresh culture and adjusted to 0.5 McFarland standard, corresponding to approximately 1 × 10^8^ CFU/mL. The suspension was subsequently diluted in MHB to obtain a final inoculum concentration of approximately 5 × 10^5^ CFU/mL in each microdilution well. The appropriate volume of the standardized bacterial inoculum was added to each well to achieve the final assay volume and inoculum concentration. MIC was defined as the lowest concentration of myricetin that completely inhibited visible bacterial growth after incubation at 37 °C for 24 h. For each myricetin concentration, a concentration-matched DMSO vehicle control containing the same final DMSO concentration as the corresponding myricetin-treated well was included to account for potential solvent effects. An untreated bacterial growth control containing MHB and the standardized bacterial inoculum, and an uninoculated MHB sterility control, were included in each assay. Streptomycin was included as a reference antibacterial control. The final bacterial inoculum concentration in each MIC well was approximately 5 × 10^5^ CFU/mL. All assays were performed in triplicate.

### 2.5. Minimum Bactericidal Concentration (MBC)

The MBC was determined followed the MIC assay. A 5 µL aliquot was collected from wells showing no visible bacterial growth and plated onto MHA plates. The plates were incubated at 37 °C for 18–24 h. The MBC was defined as the lowest concentration of myricetin that resulted in no visible colony growth on the agar plates [17].

### 2.6. Quantification of Violacein in C. violaceum

Violacein inhibition was quantified spectrophotometrically as previously described [18]. *C. violaceum* cultures were grown overnight at 30 °C in LB broth in the presence or absence of myricetin at sub-inhibitory concentrations (1/8, 1/4, and 1/2 MIC). For each myricetin concentration, a concentration-matched DMSO vehicle control containing the same final DMSO concentration as the corresponding myricetin-treated group was included. After incubation, 1 mL of culture was centrifuged at 10,000 rpm for 5 min. The pellet was resuspended in 1 mL DMSO and vortexed to extract violacein. Following centrifugation, absorbance of the supernatant was measured at 585 nm using a UV-2600 spectrophotometer (Shimadzu, Kyoto, Japan). Quercetin was used as the standard compound. Percentage of inhibition was calculated as: Violacein inhibition (%) = [(OD control − OD test)/OD control] × 100.

### 2.7. Prodigiosin Inhibition in S. marcescens

Prodigiosin production was evaluated following the method described previously [19]. *S. marcescens* (MTCC 97) was cultured in LB broth containing sub-MICs of myricetin at 30 °C (120 rpm) for 18 h. For each myricetin treatment, a concentration-matched DMSO vehicle control containing the same final DMSO concentration was included. Following centrifugation, cells were harvested, and pigments were extracted with acidified ethanol (96 mL ethanol + 4 mL 1 M HCl). After centrifugation (13,000 rpm, 5 min), absorbance of the supernatant was measured at 534 nm. Quercetin was used as the standard compound. Percentage of inhibition was calculated as: Prodigiosin inhibition (%) = [(OD control − OD test)/OD control] × 100.

### 2.8. Pyocyanin Quantification

Pyocyanin production by *P. aeruginosa* was quantified as described previously [20]. Briefly, 5 mL of bacterial culture was centrifuged at 6000× *g* for 10 min at room temperature to obtain the cell-free supernatant. The resulting 5 mL supernatant was mixed with 3 mL chloroform and vortexed for 30 s. The mixture was allowed to stand for 10 min at room temperature to facilitate phase separation. The lower chloroform phase (approximately 2 mL) containing pyocyanin was carefully transferred to a clean tube and mixed with 1 mL of 0.2 M HCl. The mixture was vortexed for 30 s and centrifuged at 6000× *g* for 2 min to facilitate phase separation. The upper acidic phase containing protonated pyocyanin was collected, and its absorbance was measured using a microplate reader. The absorbance of the acidified layer was measured at 595 nm, and inhibition percentage was calculated as: Pyocyanin inhibition (%) = [(OD control − OD test)/OD control] × 100. For each myricetin treatment concentration, a concentration-matched DMSO vehicle control containing the same final DMSO concentration was included. Quercetin was used as the standard compound.

### 2.9. Pyoverdine Measurement

Pyoverdine production was measured according to reported methods [21]. After overnight incubation with or without sub-MIC concentrations of myricetin, 5 mL of bacterial culture was centrifuged at 6000× *g* for 10 min at 4 °C to obtain a cell-free supernatant. An aliquot of 200 µL of the supernatant was transferred to a 96-well microplate and fluorescence was measured using a multimode microplate reader (BioTek, Winooski, VT, USA) at an excitation wavelength of 405 nm and an emission wavelength of 460 nm. Culture-medium blanks containing the corresponding medium without bacterial culture were included for background correction. Fluorescence values were expressed as relative fluorescence units (RFU), rather than optical density. For each myricetin treatment concentration, a concentration-matched DMSO vehicle control containing the same final DMSO concentration was included. Quercetin was used as the standard compound. Inhibition of pyoverdine production was determined as follow: Pyoverdine inhibition (%) = [(OD control − OD test)/OD control] × 100.

### 2.10. Rhamnolipid Estimation

Rhamnolipid production was measured as per previously reported method [22]. *P. aeruginosa* cultures grown with or without myricetin were incubated at 37 °C for 24 h. For each myricetin treatment concentration, a concentration-matched DMSO vehicle control containing the same final DMSO concentration was included. Following incubation, 5 mL of culture was centrifuged at 6000× *g* for 10 min to remove bacterial cells. The cell-free supernatant was extracted with an equal volume of ethyl acetate. The organic phase was collected and evaporated to dryness and the resulting extract was dissolved in 1 mL chloroform. An aliquot of 1 mL of the chloroform extract was mixed with 200 µL of 0.025% methylene blue solution and vortexed for 5 min. The mixture was allowed to stand for 15 min for phase separation. The chloroform phase was then transferred to a clean tube containing 500 µL of 0.2 N HCl and vortexed for 20 s. After phase separation, 200 µL of the upper acidic phase was transferred to a 96-well microplate, and absorbance was measured at 638 nm. A 0.2 N HCl blank was used for background correction. Rhamnolipid production was expressed relative to the corresponding control, and inhibition was calculated as: Rhamnolipid inhibition (%) = [(OD control − OD test)/OD control] × 100. Quercetin was used as the standard compound.

### 2.11. Biofilm Formation Inhibition Assay

The effect of myricetin on biofilm formation was evaluated using a crystal violet-based tube assay as previously described with minor modification [23]. Briefly, 3 mL of MHB supplemented with 1% glucose was dispensed into sterile glass tubes, followed by the addition of 250 µL of standardized bacterial inoculum and 250 µL of myricetin at sub-inhibitory concentrations (1/8, 1/4, and 1/2 MIC). Control tubes received the corresponding volume of DMSO vehicle, with the final DMSO concentration matched to that present in the corresponding myricetin-treated tubes. Following incubation at 37 °C for 72 h under shaking conditions (120 rpm), the planktonic cells were discarded and the tubes were gently washed with phosphate-buffered saline (PBS) to remove non-adherent cells. The attached biofilms were stained with 0.1% crystal violet for 15 min, washed thoroughly with distilled water, and air-dried. The bound dye was then solubilized using 33% acetic acid and absorbance was measured at 595 nm. Quercetin was used as the standard compound. Because myricetin was present during the entire biofilm-development period, this assay was designed to assess inhibition of biofilm formation rather than disruption or eradication of a pre-established mature biofilm. Reduction in total attached biofilm biomass was expressed as percentage reduction relative to the corresponding control and calculated as follows: Biofilm biomass reduction (%) = [(OD control − OD test)/OD control] × 100.

### 2.12. Extraction and Quantification of Extracellular Polymeric Substances (EPS)

EPS production was quantified as per previously described method [24] with minor modifications. Bacterial cultures were grown in the presence or absence of sub-inhibitory concentrations of myricetin and incubated at 37 °C for 18 h. For each myricetin treatment concentration, a concentration-matched DMSO vehicle control containing the same final DMSO concentration was included. Following incubation, cultures were centrifuged at 6000× *g* for 10 min at 4 °C and the cell pellets were collected. The pellets were gently resuspended in 2 mL of high-salt buffer (0.5 M NaCl, 10 mM Tris-HCl, pH 7.5) and incubated for 30 min at room temperature with gentle agitation to facilitate EPS release. The suspensions were subsequently centrifuged at 10,000× *g* for 15 min at 4 °C, and the supernatants containing extracted EPS were carefully collected. EPS was precipitated by adding three volumes of chilled 95% ethanol and incubating the mixture at −20 °C for 12 h. The precipitated EPS was recovered by centrifugation at 10,000× *g* for 15 min at 4 °C and dissolved in 1 mL of Milli-Q water. For carbohydrate estimation, 200 µL of the EPS solution was mixed with 200 µL of 5% phenol, followed by addition of 1 mL concentrated sulfuric acid. The mixture was incubated at room temperature for 30 min, and absorbance was measured at 490 nm. A reagent blank containing Milli-Q water and all reagents without EPS extract was included for background correction. Quercetin was used as the standard compound. EPS inhibition was express as percentage inhibition and calculated using the following formula: EPS inhibition (%) = [(OD control − OD test)/OD control] × 100.

### 2.13. Molecular Docking Analysis

Molecular docking analysis was performed to investigate the interaction of myricetin with selected quorum sensing and biofilm-associated target proteins. Three-dimensional structures of the target proteins were retrieved from the RCSB Protein Data Bank (PDB) [25]. Prior to docking, protein structures were prepared by removing water molecules, co-crystallized ligands and other heteroatoms using PyMOL software (version 2.5) [26]. Molecular docking was done with AutoDock Vina engine (Version 1.1.2) available in PyRx 0.8. The protein structures used in this study were obtained experimentally from the RCSB Protein Data Bank and included the following: LasI (PDB ID: 1RO5, Chain A), LasR (2UV0, Chain E, PilY1 (3HX6, Chain A), LasA (3IT7, Chain A), PilT (3JVV, Chain A), CviR (3QP5, Chain A), PqsR (4JVI, Chain A), EsaI (Esal, Chain A) and CviR (3QP1, Chain A). Each structure was checked prior to preparation of receptors and only the chain needed for docking was kept. The receptor was prepared with polar hydrogen atoms, AutoDock atom types and Gasteiger partial charges, by using the functionality provided by AutoDockTools within PyRx 0.8. The proteins were then converted to rigid-receptor PDBQT files. Protonation states were assigned during receptor preparation, and the protein was treated as a rigid receptor during docking. The hydrogen atoms added in the preparation of the receptor were chosen to represent protonation. The three-dimensional structure of myricetin (PubChem CID 4276) was acquired via the built-in Open Babel module and loaded into PyRx. Myricetin was modeled in the neutral form (Formal charge 0) because it is lacking in a strongly ionizable functional group at physiological pH. The hydrogen atoms were added, the bond orders and aromaticity were evaluated, and the geometry of the ligand was energy minimized using Universal Force Field. The conjugate-gradient algorithm was used for the minimization of the energy, to a maximum of 200 steps or until the energy change per step was smaller than 0.1 kcal mol^−1^. The minimized ligand was then converted to the AutoDock-compatible PDBQT format, and Gasteiger partial charges and rotatable bonds were assigned using the PyRx/Open Babel preparation workflow [27]. The receptor was treated as rigid while all the torsions of the ligand were allowed to rotate during docking. A structurally relevant co-crystallized ligand was used for both targeted and blind-docking approaches. The search space was focused on the centroid of the heavy atoms of the native ligand, excluding the atoms of the receptor. The LasR/2UV0 box was centered on at x = 23.0166, y = 15.8588, and z = 80.0661 Å; the LasA/3IT7 box was centered on at x = 13.0106, y = 5.7986, and z = −8.5838 Å; the PilT/3JVV box was centered at x = −15.8387, y = 21.6480, and z = 28.0392 Å; the CviR/3QP5 box was centered at x = 30.6521, y = 12.0599, and z = −4.7972 Å; and the PqsR/4JVI box was centered at x = −33.4890, y = 56.8654, and z = 9.1002 Å. For each of the targeted systems a search-space of 25 × 25 × 25 Å was used and inspected visually to ensure that the entire native-ligand pocket and surrounding residues were covered. The LasI blind-docking grid was centered at x = 41.1284, y = −10.4161, and z = −13.6051 Å, with dimensions of 50.7909 × 45.8581 × 46.1983 Å. The PilY1/3HX6 grid was centered at x = 8.0362, y = 8.6483, and z = 6.9969 Å, with dimensions of 66.4352 × 64.8285 × 55.0055 Å. For EsaI (PDB ID: 1KZF, chain A), grid center of x = 23.7475, y = −1.6435, and z = 15.4295 Å, with dimensions of 52.827 × 60.447 × 54.101 Å. The (PDB ID: 3QP1, chain A), x = 20.3781 Å, y = 12.8896 Å, and z = 48.7434 Å with the dimensions of the grid were set to 25 × 25 × 25 Å. The Vina scoring function with a maximum of 9 output poses (num_modes = 9) and energy range of 3 kcal mol^−1^ was used to perform docking calculations. To assess search convergence, at least three separate docking calculations were carried out for each receptor–ligand system using different random seeds. The docking poses generated were automatically clustered and ranked in order of predicted docking scores, which were generated by AutoDock Vina in kcal mol^−1^. The software BIOVIA Discovery Studio Visualizer (v21.1.0.20298) and PyMOL were used to visualize the binding modes and interactions. Docking validation was additionally performed for targets containing experimentally determined co-crystallized ligands by removing the native ligand and subsequently redocking it into the corresponding binding pocket using the same docking protocol. The RMSD between the crystallographic and redocked native-ligand poses was calculated. These RMSD values were used to assess the ability of the docking protocol to reproduce experimentally observed binding modes. Molecular docking scores were interpreted as computational estimates of ligand-protein affinity and were not considered experimental evidence of direct target engagement.

### 2.14. Molecular Dynamics Simulation

CviR (3QP5) and LasR (2UV0) were selected from the docked targets because they showed the most favorable predicted binding affinities and represent well-characterized QS regulatory receptors relevant to the QS-associated phenotypes investigated experimentally. The stability and conformational dynamics of the top-ranked myricetin–protein complexes were further evaluated using all-atom molecular dynamics (MD) simulations. The same verified myricetin ligand structure used in the docking calculations was retained for the subsequent molecular dynamics simulations. The ligand identity and chemical structure were maintained consistently throughout the docking, system preparation, molecular dynamics simulations, trajectory analysis, PCA, DCCM, protein–ligand interaction analysis and MM-GBSA calculations. Complexes corresponding to PDB IDs 3QP5 and 2UV0 were selected for simulation using the Desmond software package ((version 2019-4; Schrodinger LLC, New York, NY, USA) [28]. Each system was solvated in an orthorhombic simulation box using the TIP3P explicit water model, maintaining a buffer distance of 10 Å between the solute and the box boundaries [29]. Counter ion (Na^+^ or Cl^−^) were added to neutralize the systems, and the ionic strength was adjusted to 0.15 M NaCl. The systems were parameterized using the OPLS_2005 force field and subjected to energy minimization and equilibration steps to remove steric clashes and stabilize the systems [30]. Production simulations were carried out for 100 ns under NPT conditions at 310 K and 1 atm using the Nose-Hoover thermostat and Martyna-Tobias-Klein barostat [31]. Trajectory analysis included root mean square deviation (RMSD), root mean square fluctuation (RMSF), secondary structure elements (SSE) and protein–ligand interaction profiling using the Simulation Interaction Diagram (SID) tool. Principal component analysis (PCA) was performed using the Bio3D package in R (version 2.4-5) [32]. Binding free energy of the myricetin–protein complexes was further estimated using the Molecular Mechanics-Generalized Born Surface Area (MM-GBSA) method implemented in the Schrodinger software package (Release 2019-4; Schrödinger LLC, New York, NY, USA) [33]. MM-GBSA analysis was performed on snapshots extracted from the 100 ns MD production trajectories to estimate the binding free energies of the myricetin–protein complexes. The calculated values were interpreted as approximate binding-energy estimates rather than quantitative measures of thermodynamic stability. No explicit entropy calculation was performed. MM-GBSA calculations were performed to quantitatively quantify the thermodynamic stability of the selected lead compounds in the active site. The thermal snapshots of the protein–ligand complexes were retrieved from the 100 ns MD production trajectories. The following fundamental thermodynamic Equation (1) was used to calculate the binding free energy ∆G_binding.

∆Gbind = Gcomplex − (Gprotein + Gligand) (1)

∆Gbind represents the binding free energy, Gcomplex represents the complex free energy, Gprotein represents the target protein, and Gligand represents the ligand’s free energy.

### 2.15. Statistical Analysis

All experiments were performed using three independent biological experiments, and results are expressed as mean ± standard deviation (SD). Statistical analysis of antibacterial activity was performed using two-way analysis of variance (ANOVA), followed by Bonferroni multiple comparisons. For the other assays, one-way ANOVA followed by Dunnett’s post hoc test was used for comparisons with the designated reference group. A *p* value < 0.05 was considered statistically significant. Statistical analyses were performed using GraphPad Prism (version 8.0; GraphPad Software, Inc., San Diego, CA, USA).

## 3. Results

### 3.1. Antibacterial Activity of Myricetin

**Figure 1:**
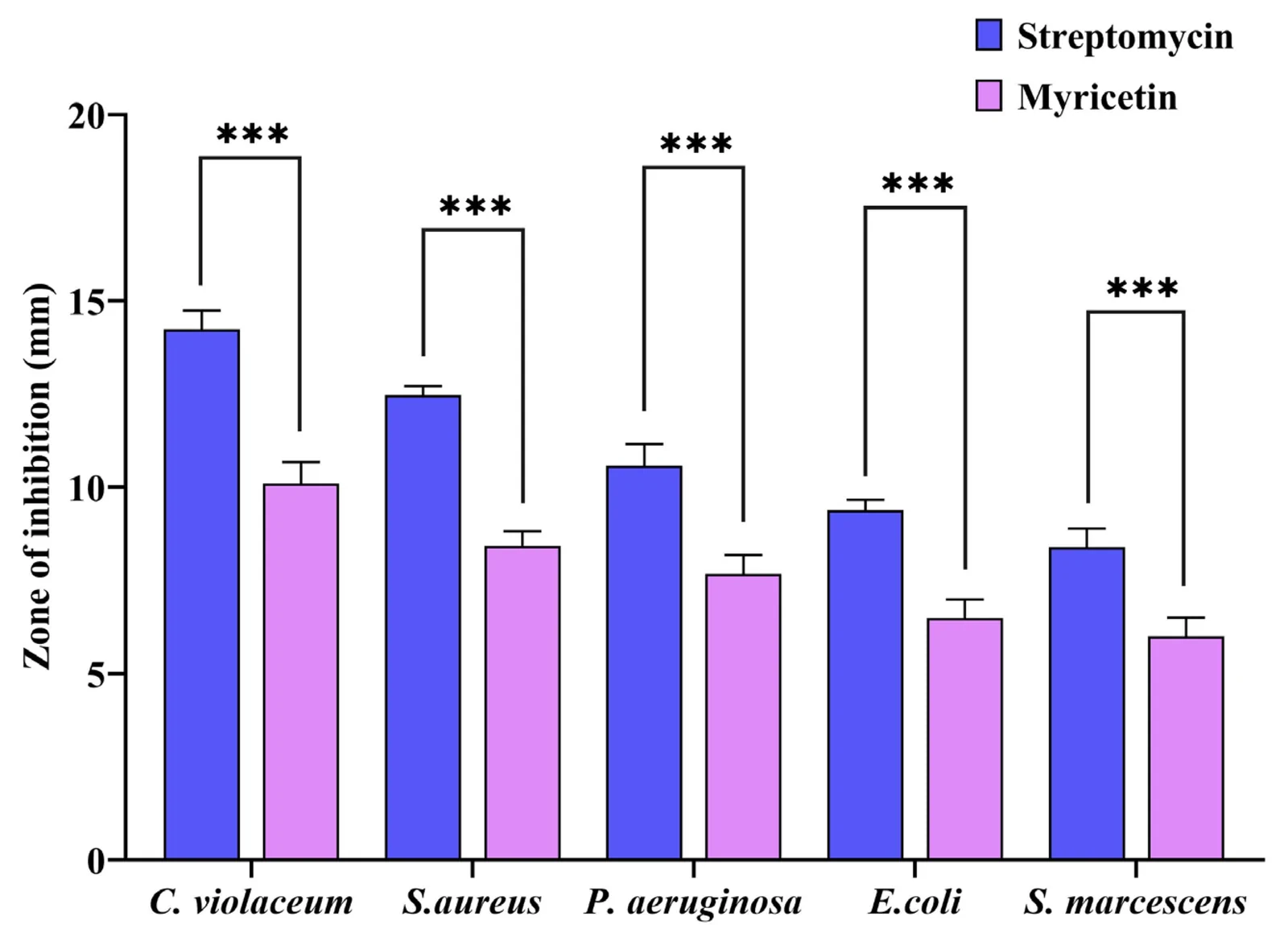
Assessment of the antibacterial activity of myricetin in comparison with streptomycin against selected bacterial pathogens. Data are expressed as mean ± standard deviation (SD) derived from three independent experiments. All values are presented as mean ± SD of three independent experiments. Statistical significance is indicated as: (*** *p* < 0.0005).

Myricetin exhibited antibacterial activity against all tested bacterial strains in the agar well diffusion assay. Among the tested strains, *Chromobacterium violaceum* showed the highest susceptibility, producing the largest inhibition zone, followed by *Staphylococcus aureus*, *Pseudomonas aeruginosa*, *Escherichia coli* and *Serratia marcescens* (Figure 1).

MIC values determined by broth microdilution were 250 µg/mL for *S. aureus* and *C. violaceum*, 500 µg/mL for *E. coli* and *P. aeruginosa*, and 1000 µg/mL for *S. marcescens*. MBC values were 500 µg/mL for *C. violaceum* and *S. aureus*, 1000 µg/mL for *P. aeruginosa* and *E. coli*, whereas *S. marcescens* exhibited an MBC value >1000 µg/mL. Subsequent anti-virulence and biofilm formation inhibition assays were conducted at sub-MIC levels (1/2, 1/4, and 1/8 MIC) to evaluate QS-associated and biofilm-related phenotypes while minimizing the influence of direct growth inhibition. Growth assessment detected no statistically significant differences at the tested sub-MICs (*p* > 0.05). However, in the absence of complete time-course growth curves, growth-related contributions to the observed phenotypic reductions cannot be conclusively excluded.

### 3.2. Effect of Sub-MIC Concentrations and DMSO Vehicle Controls on Bacterial Growth

To determine whether the concentrations used for the anti-QS, anti-virulence and biofilm formation inhibition assays affected bacterial growth, the growth of all tested bacterial strains was evaluated in the presence of myricetin at 1/8, 1/4 and 1/2 MIC together with their corresponding concentration-matched DMSO vehicle controls. No significant differences in bacterial growth were observed between the untreated controls and the corresponding DMSO vehicle controls (*p* > 0.05), indicating that the solvent concentrations used in the experiments did not significantly affect bacterial growth. Similarly, the myricetin-treated groups at the tested sub-MICs showed no significant reduction in bacterial growth compared with their corresponding vehicle controls (*p* > 0.05). These findings support the interpretation that the effects observed in subsequent QS, virulence-factor and biofilm assays were not primarily attributable to DMSO-mediated growth inhibition.

### 3.3. Suppression of QS-Controlled Pigment in C. violaceum

**Figure 2:**
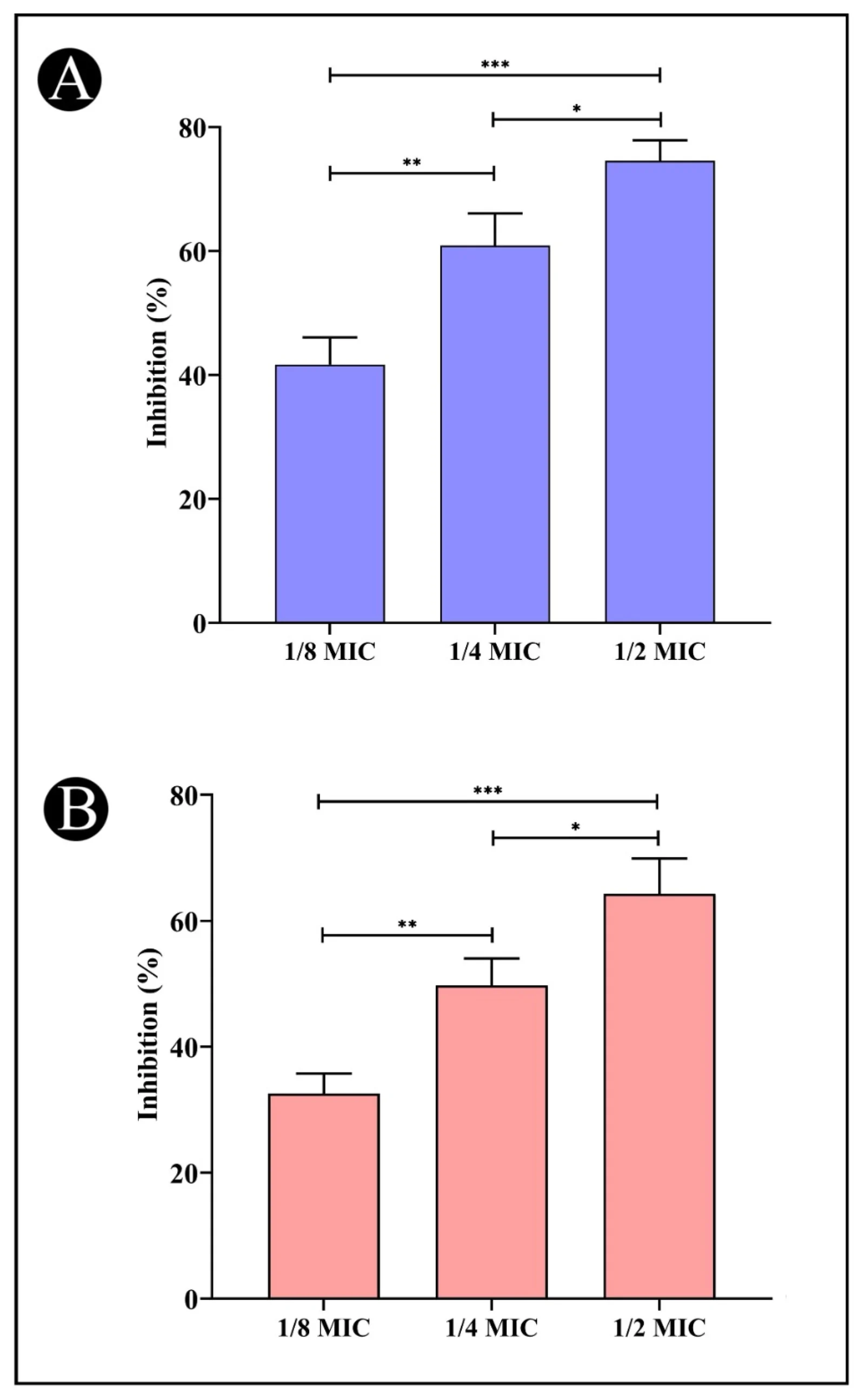
(**A**) Quantitative evaluation of violacein production in *C. violaceum* following treatment with myricetin. (**B**) Quantitative assessment of prodigiosin production in *S. marcescens* under myricetin exposure. All values are presented as mean ± SD of three independent experiments. Statistical significance is indicated as: (* *p* < 0.05, ** *p* < 0.005, and *** *p* < 0.0005).

The effect of myricetin on the QS-associated pigment violacein was evaluated using *C. violaceum*, a well-established QS reporter strain. Pigment production decreased in a concentration-dependent manner. At 1/8, 1/4, and 1/2 MIC, violacein levels were reduced by myricetin by 41.69%, 60.80%, and 74.59%, respectively (Figure 2A). These findings indicate concentration-dependent modulation of a QS-associated phenotype under the tested conditions.

### 3.4. Attenuation of Prodigiosin in S. marcescens

The effect of myricetin on the QS-associated production of prodigiosin was further evaluated in *S. marcescens*. Sub-MICs of myricetin significantly reduced this QS-regulated pigment in a concentration-dependent manner. At 1/8, 1/4, and 1/2 MIC, prodigiosin production was inhibited by 32.58%, 49.74%, and 64.32%, respectively (Figure 2B). These findings are consistent with potential QS-modulatory activity of myricetin in *S. marcescens* under the tested conditions (*p* < 0.05).

### 3.5. Modulation of P. aeruginosa Virulence Factors

**Figure 3:**
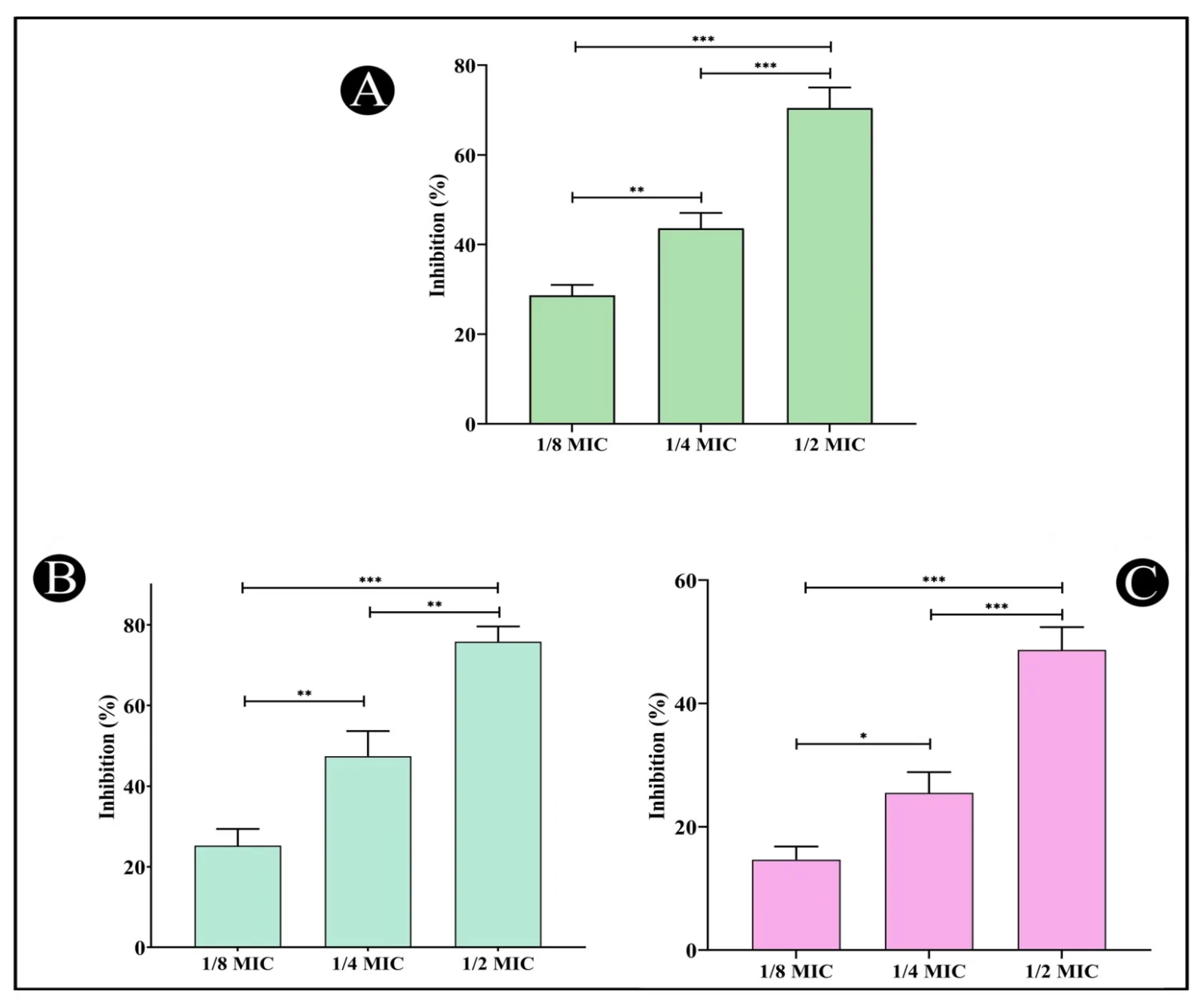
(**A**) Quantitative measurement of pyocyanin production in *P. aeruginosa* in the presence of myricetin. (**B**) Quantification of pyoverdine levels in *P. aeruginosa* following treatment with myricetin. (**C**) Determination of rhamnolipid production in *P. aeruginosa* under myricetin treatment. Results are expressed as mean ± SD from three independent experiments. Statistical significance is represented as: (* *p* < 0.05, ** *p* < 0.005, and *** *p* < 0.0005).

The effect of myricetin on QS-dependent virulence factors in *P. aeruginosa* was evaluated. Pyocyanin production was significantly reduced in a concentration-dependent manner, with inhibition rates of 28.67%, 43.58%, and 70.44% at 1/8, 1/4, and 1/2 MIC, respectively (Figure 3A). Similarly, pyoverdine synthesis showed a marked decrease, with inhibition rates of 25.22%, 47.43%, and 75.84% at corresponding concentrations (Figure 3B). Rhamnolipid production was also reduced, with decreases of 14.67%, 25.49%, and 48.69% observed across increasing sub-MIC levels (Figure 3C). These results indicate a significant reduction in virulence factors production in *P. aeruginosa* (*p* < 0.05).

### 3.6. Inhibition of Biofilm Formation

**Figure 4:**
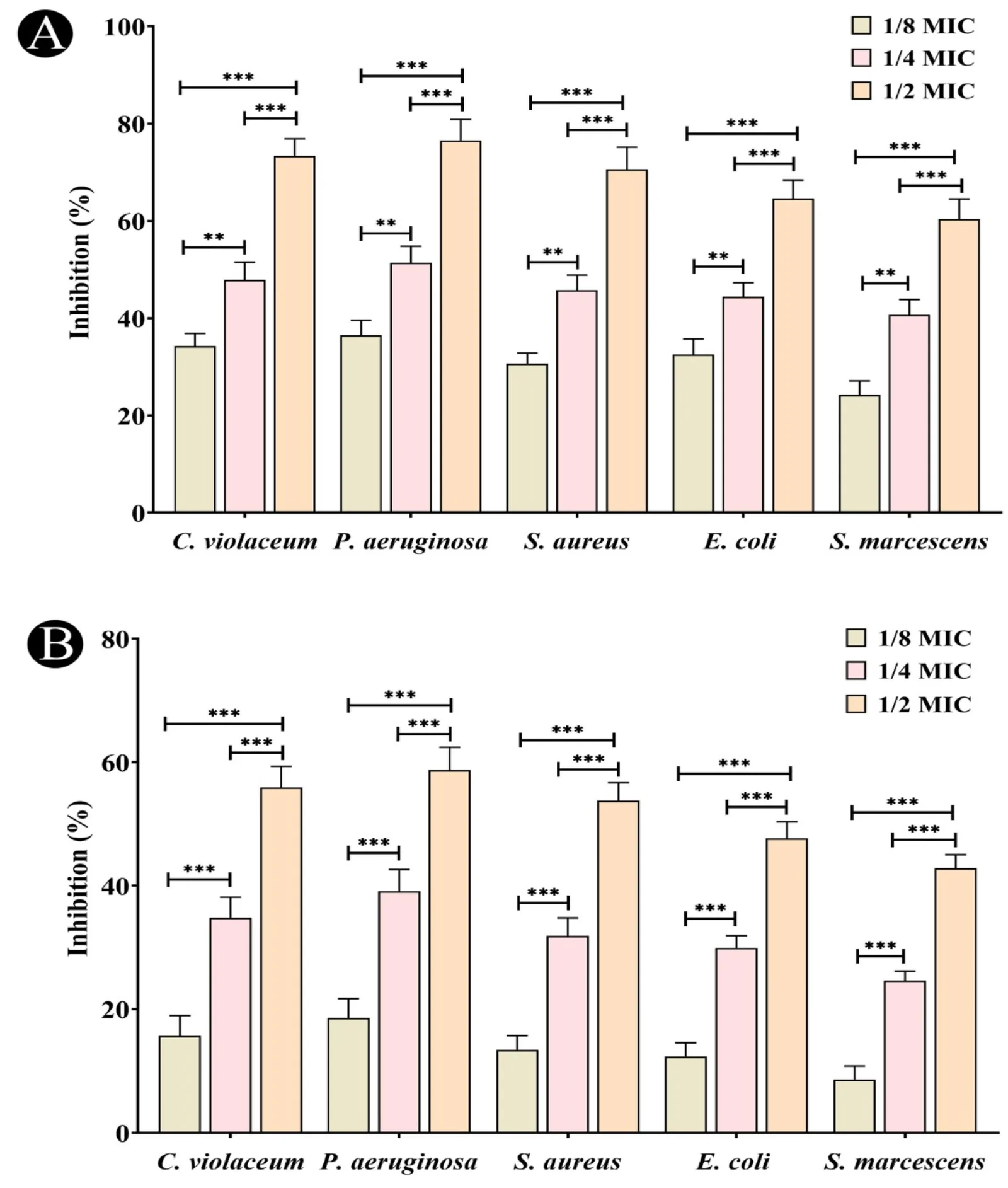
Effect of myricetin on biofilm formation and extracellular polymeric substance (EPS) production. (**A**) Inhibition of biofilm formation, quantified as the reduction in total attached biomass by crystal violet staining, with myricetin present during biofilm development. This assay did not assess disruption of established biofilms or killing of biofilm-associated cells. (**B**) Quantitative estimation of EPS reduction upon myricetin treatment. Data are shown as mean ± SD of three independent experiments. Statistical significance is denoted as: (** *p* < 0.005, and *** *p* < 0.0005).

The effect of myricetin on biofilm formation was evaluated against all tested bacterial strains, revealing a concentration-dependent inhibition of biofilm formation (Figure 4A). Sub-MICs of myricetin significantly reduced biofilm biomass across all organisms examined. In *C. violaceum*, biofilm formation was inhibited by 34.28%, 47.83%, and 73.38% at 1/8, 1/4, and 1/2 MIC, respectively. Similarly, *P. aeruginosa* exhibited reductions of 36.49%, 51.38%, and 76.54% at corresponding concentrations. In *S. aureus*, biofilm biomass decreased by 30.66%, 45.76%, and 70.59%, while *E. coli* showed inhibition levels of 32.53%, 44.39%, and 64.59%. In comparison, *S. marcescens* exhibited relatively lower, but significant reductions of 24.22%, 40.69%, and 60.38% under the same conditions. These findings demonstrate that myricetin inhibits biofilm formation and reduces total attached biofilm biomass under the tested experimental conditions (*p* < 0.05).

### 3.7. Reduction in EPS Production

The effect of myricetin on EPS production was quantitatively assessed across all tested bacterial strains. As shown in Figure 4B, myricetin treatment resulted in a concentration-dependent reduction in EPS levels. In *C. violaceum*, EPS production decreased by 15.73%, 34.82%, and 55.90% at 1/8, 1/4, and 1/2 MIC, respectively. A similar trend was observed in *P. aeruginosa*, with reduction of 18.58%, 39.10%, and 58.72%. In *S. aureus*, ESP levels were reduced by 13.48%, 31.88%, and 53.77%, while *E. coli* showed decreases of 12.39%, 29.93%, and 47.67%. In comparison, *S. marcescens* exhibited relatively lower but still significant reductions of 8.66%, 24.64%, and 42.81% under the same conditions. These results demonstrate that myricetin significantly reduces EPS production across multiple bacterial species in a concentration-dependent manner (*p* < 0.05).

### 3.8. Molecular Docking Analysis

**Figure 5:**
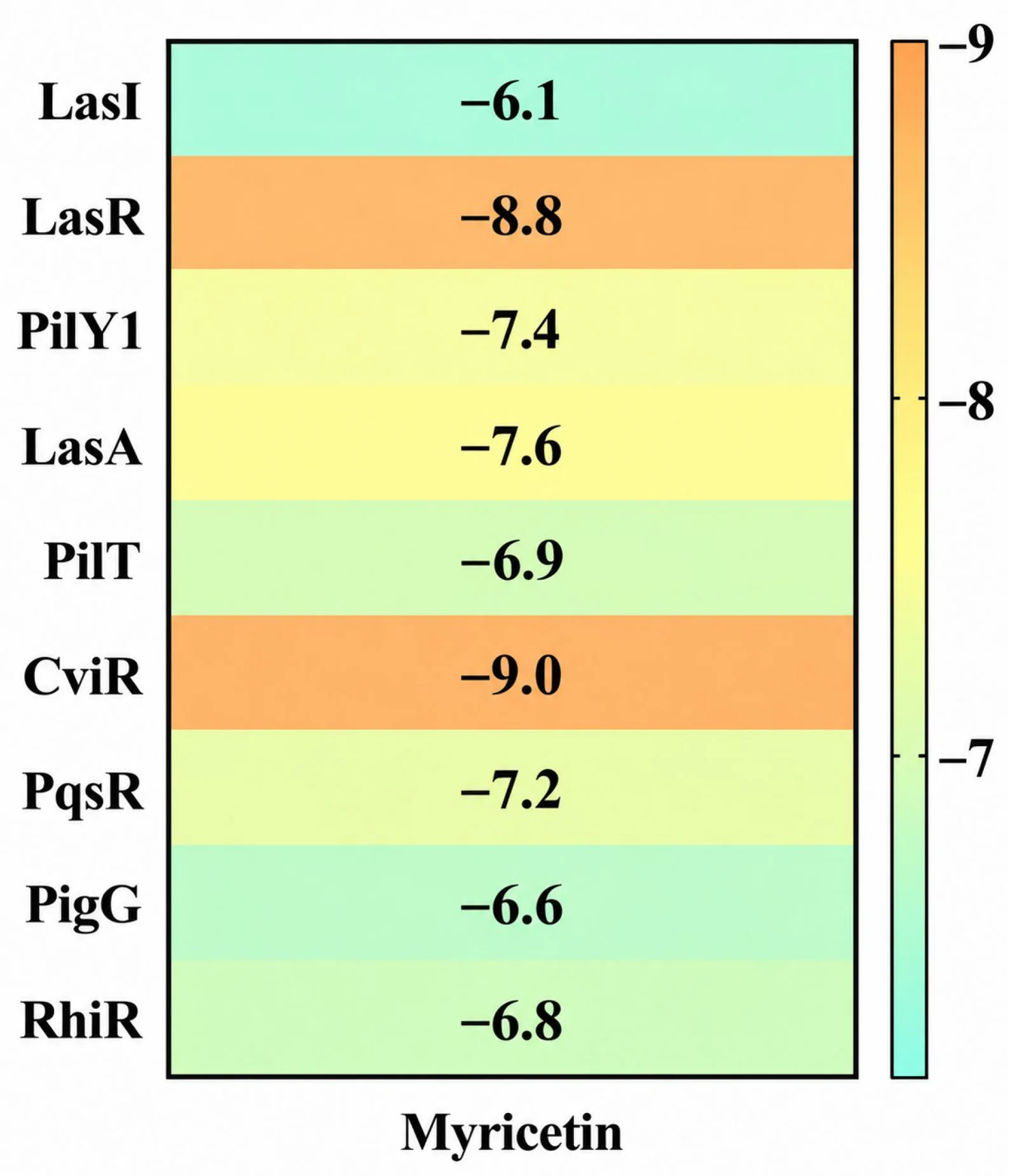
Heatmap representation illustrating the binding affinities of myricetin against selected QS and biofilm-associated target proteins. The color gradient reflects the strength of interaction, where more negative binding energy values indicate stronger ligand–protein affinity. This visualization highlights the top-ranked targets exhibiting the most favourable binding interactions with myricetin.

**Figure 6:**
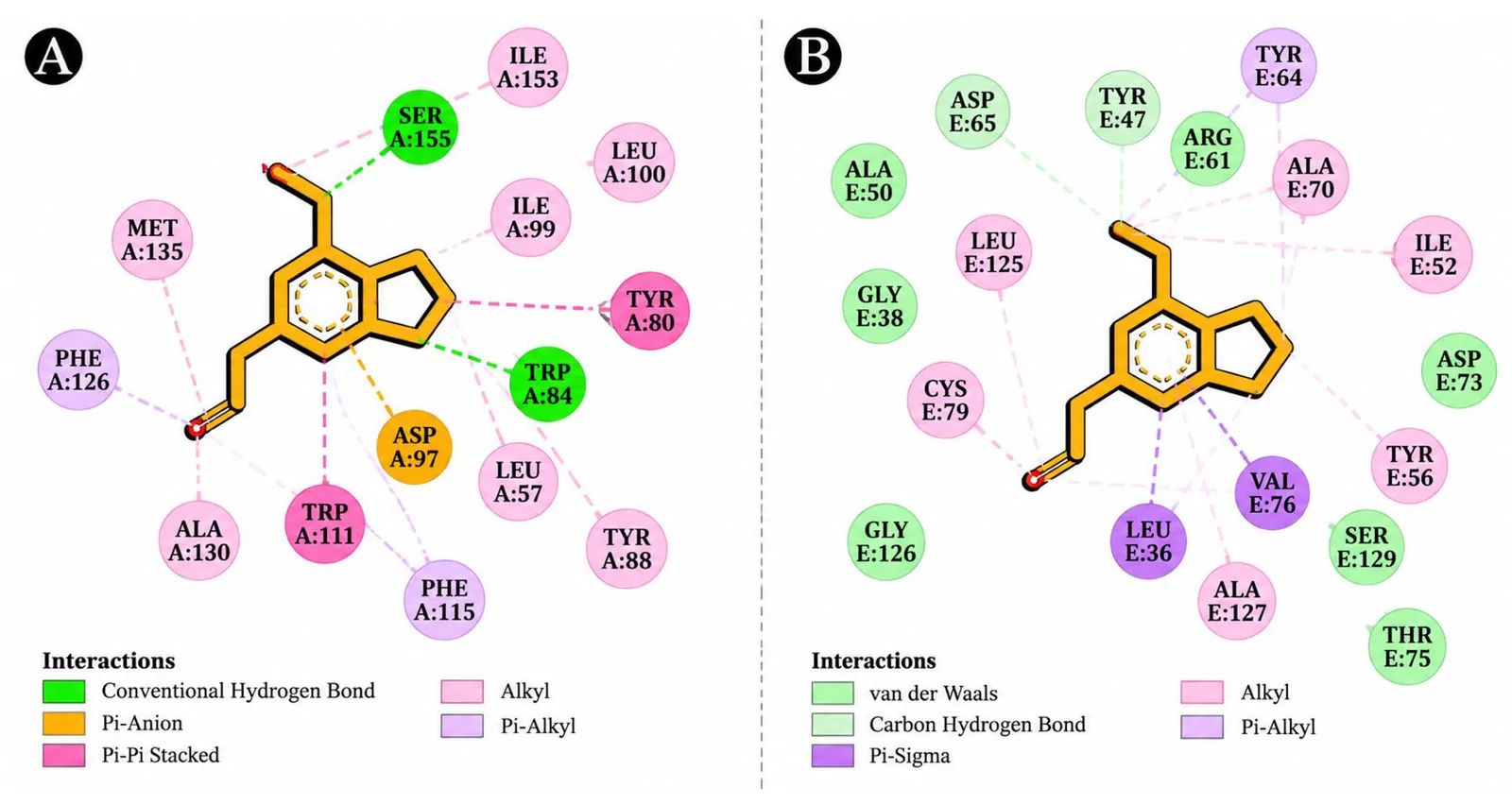
Interaction profiles depicting the binding interfaces and molecular docking interactions of the top-ranked Myricetin–protein complexes. (**A**) 3QP5–myricetin complex showing key hydrogen bonding, π–π stacking, and hydrophobic interactions within the binding pocket. (**B**) 2UV0–myricetin complex illustrating the network of stabilizing interactions, including hydrogen bonds, π-based interactions, and hydrophobic contacts that facilitate ligand accommodation and stability.

All computational analyses described below were performed using myricetin as the ligand. The ligand identity was verified against the corresponding chemical structure and computational input file and the same myricetin structure was used throughout molecular docking and subsequent molecular dynamics and trajectory-based analyses. Molecular docking simulations were performed to evaluate the binding affinity of myricetin toward selected quorum sensing-related target proteins. The results revealed binding energies ranging from −9.0 to −6.1 kcal/mol, indicating variable ligand-protein interactions across the targets. Among the evaluated protein, CviR (PDB ID: 3QP5) exhibited the highest binding affinity (−9.0 kcal/mol), followed by LasR (PDB ID: 2UV0) with a binding energy of −8.8 kcal/mol (Figure 5). Based on these results, both complexes were selected for further MD simulations. The selection of CviR and LasR for subsequent MD simulations was based on their top-ranked docking scores and their established roles as key LuxR-family QS regulatory receptors, representing QS systems associated with the phenotypic responses evaluated in this study. In the 3QP5 complex, myricetin was positioned within a hydrophobic binding pocket and formed key hydrogen bonds with Trp84 and Ser155. Additional interactions, including π–π stacking with Tyr80 and hydrophobic contacts with surrounding residues, contributed to ligand stabilization (Figure 6A). Similarly, in the 2UV0 complex, myricetin formed hydrogen bonds with Tyr47 and Asp65, along with hydrophobic and π–alkyl interactions that supported stable binding within the active site (Figure 6B). These results support the predicted favourable interaction of myricetin with the selected QS regulatory proteins.

### 3.9. MD Simulation Analysis

To evaluate the dynamic stability of the top-ranked docked complexes, 100 ns MD simulations were performed for the 3QP5-myricetin complex and 2UV0-myricetin complex.

#### 3.9.1. Structural Stability Analysis (RMSD)

**Figure 7:**
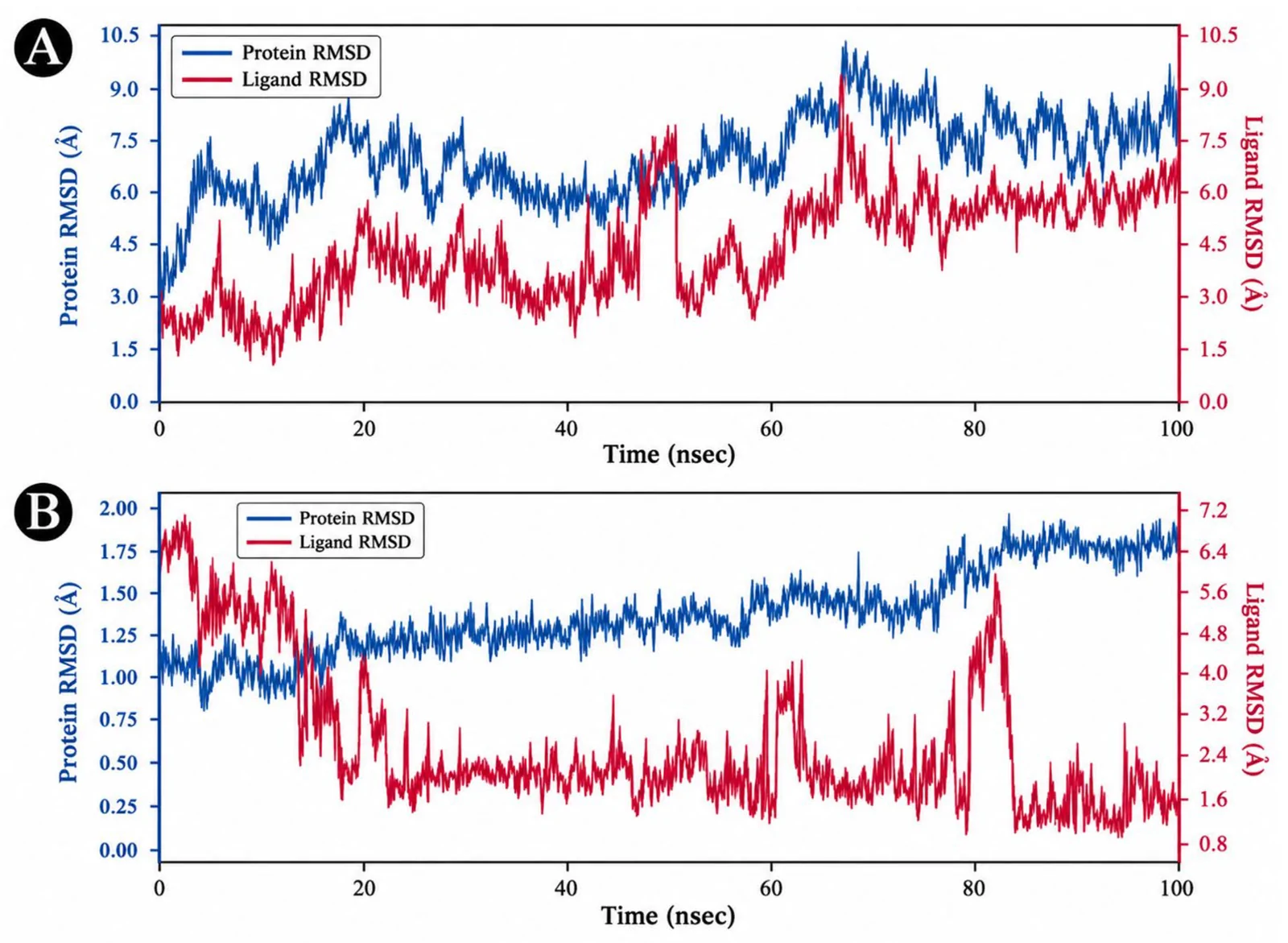
Time evolution of root mean square deviation (RMSD) profiles of the protein Cα backbone and bound ligand over a 100 ns molecular dynamics simulation. (**A**) 2UV0–myricetin complex and (**B**) 3QP5–myricetin complex. Both systems reached relatively stable RMSD profiles after the initial equilibration phase, indicating maintenance of the overall structural state of the protein–ligand complexes under the simulated conditions.

The root mean square deviation (RMSD) profiles of protein backbones and ligands indicated stable system equilibration throughout the simulation (Figure 7A,B). The 3QP5-myricetin complex exhibited an initial equilibration phase within the first 20 ns, after which the RMSD values remained within the range of 2–3 Å, indicating preservation of the overall protein structure without major conformational changes. The ligand RMSD stabilized following equilibration, with minor fluctuations observed around 50 ns, suggesting slight positional adjustments within the binding pocket while maintaining stable binding. In contrast, the 2UV0-myricetin complex showed rapid convergence, with protein RMSD stabilizing early and exhibiting minimal fluctuations throughout the simulation. The ligand RMSD remained stable after approximately 20 ns, with only transient fluctuations attributed to rotational flexibility. Overall, both systems exhibited stable behavior, with the 2UV0 complex showing slightly lower fluctuations, indicating marginally higher dynamic stability.

#### 3.9.2. Residue Flexibility Analysis (RMSF)

**Figure 8:**
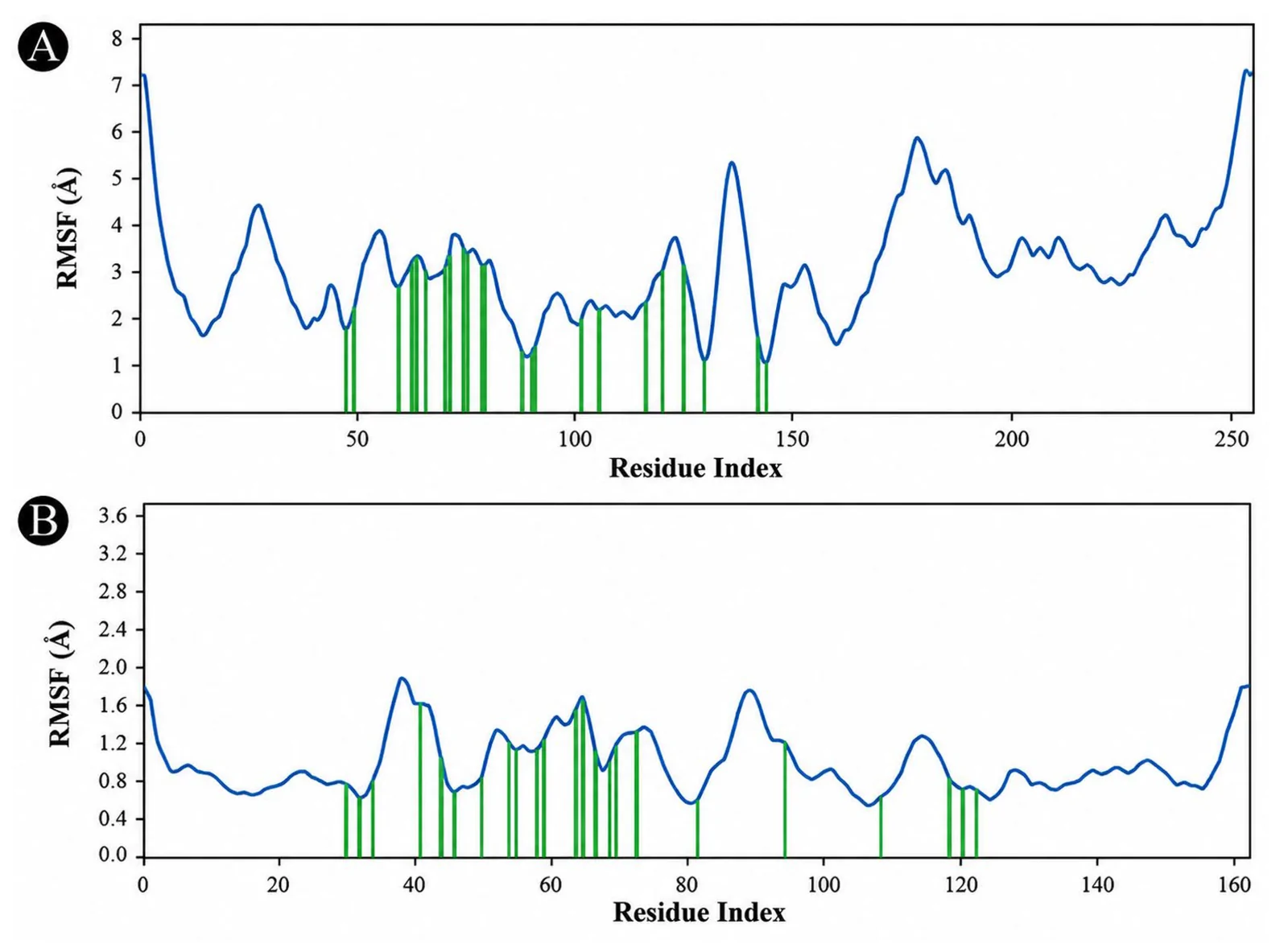
Root means square fluctuation (RMSF) profiles of receptor residues in complex with myricetin: (**A**) 2UV0 and (**B**) 3QP5. The profiles show the residue-level flexibility observed during the 100 ns simulations, with relatively lower fluctuations in the binding-site regions and higher fluctuations in loop and terminal regions. Green vertical lines indicate residues involved in the ligand-binding region. Relatively lower fluctuations are observed in the binding-site regions, whereas higher fluctuations occur primarily in loop and terminal regions.

The root mean square fluctuation (RMSF) indicated limited flexibility within the core regions of both proteins (Figure 8A,B). In the 3QP5–myricetin complex, α-helices and β-sheets exhibited minimal fluctuations, while higher mobility was observed in loop regions and terminal residues, as expected for solvent-exposed domains. Notably, residues within the binding pocket showed reduced fluctuations, indicating stable ligand accommodation. Similarly, the 2UV0–myricetin complex demonstrated low RMSF values across the protein backbone, particularly within the active site residues, reflecting a stable binding environment. These results indicate that the residues of both protein–myricetin complexes exhibited relatively limited fluctuations within the simulated trajectories, particularly around the binding-site regions.

#### 3.9.3. Secondary Structure Stability (SSE)

**Figure 9:**
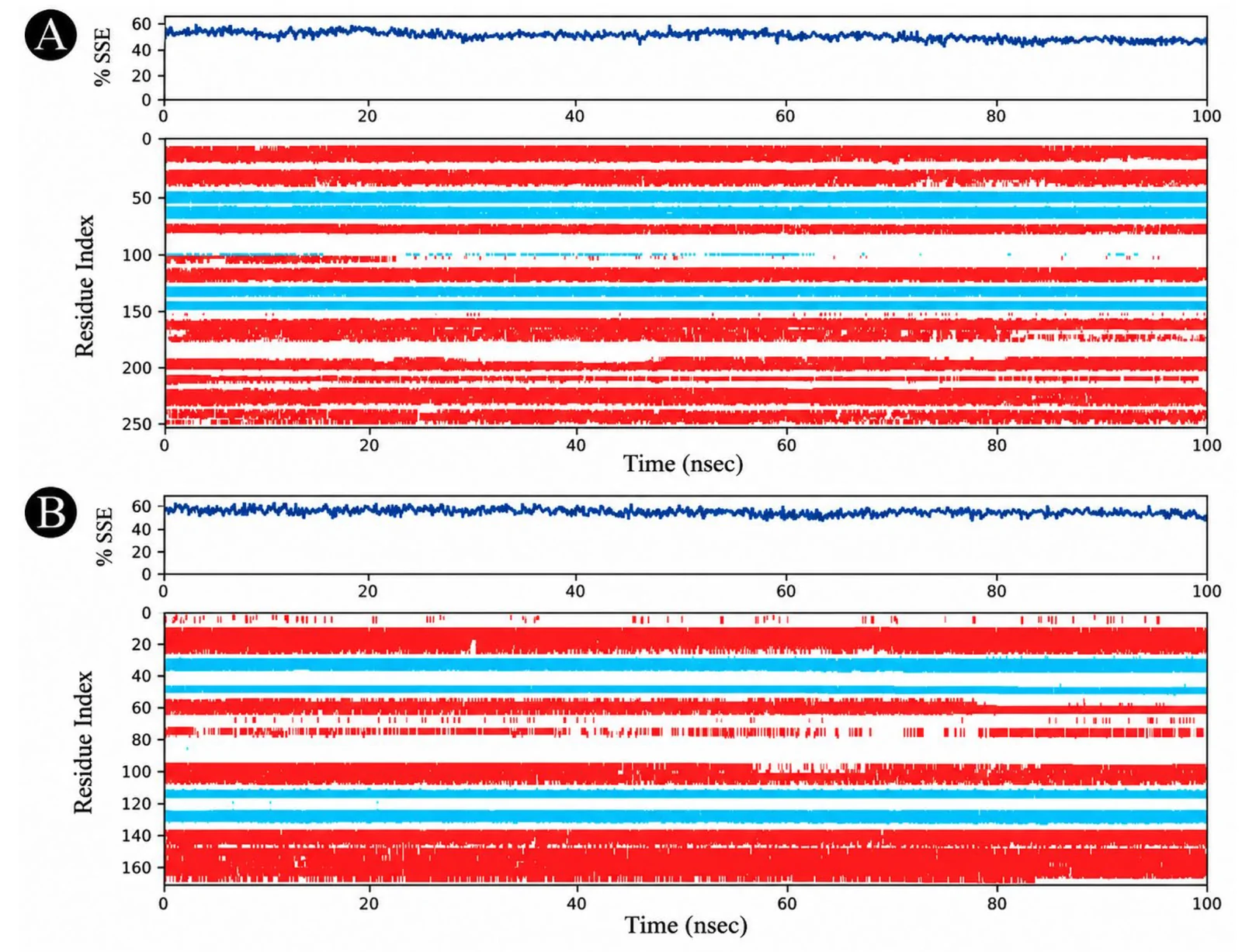
Time-resolved analysis of secondary structure elements (SSE) during the 100 ns simulation for (**A**) 2UV0–myricetin and (**B**) 3QP5–myricetin complexes. The consistent distribution of α-helices and β-strands throughout the trajectory indicates preservation of the native protein architecture and indicating preservation of the overall secondary structural architecture throughout the simulations. Different colors represent different secondary structure elements as assigned by the SSE analysis.

Secondary structure element (SSE) analysis indicated that the secondary structures of both protein–myricetin complexes remained relatively stable throughout the simulations (Figure 9A,B). The 3QP5–myricetin complex maintained a total SSE content of 53.26%, comprising 39.74% α-helices and 13.52% β-strands, which remained stable throughout the simulation. Similarly, the 2UV0–myricetin complex retained a stable SSE composition (total 51.62%; helices 37.96%; strands 13.67%). These results indicate preservation of the overall secondary structural architecture during the simulated trajectories.

#### 3.9.4. Protein–Ligand Interaction Analysis

**Figure 10:**
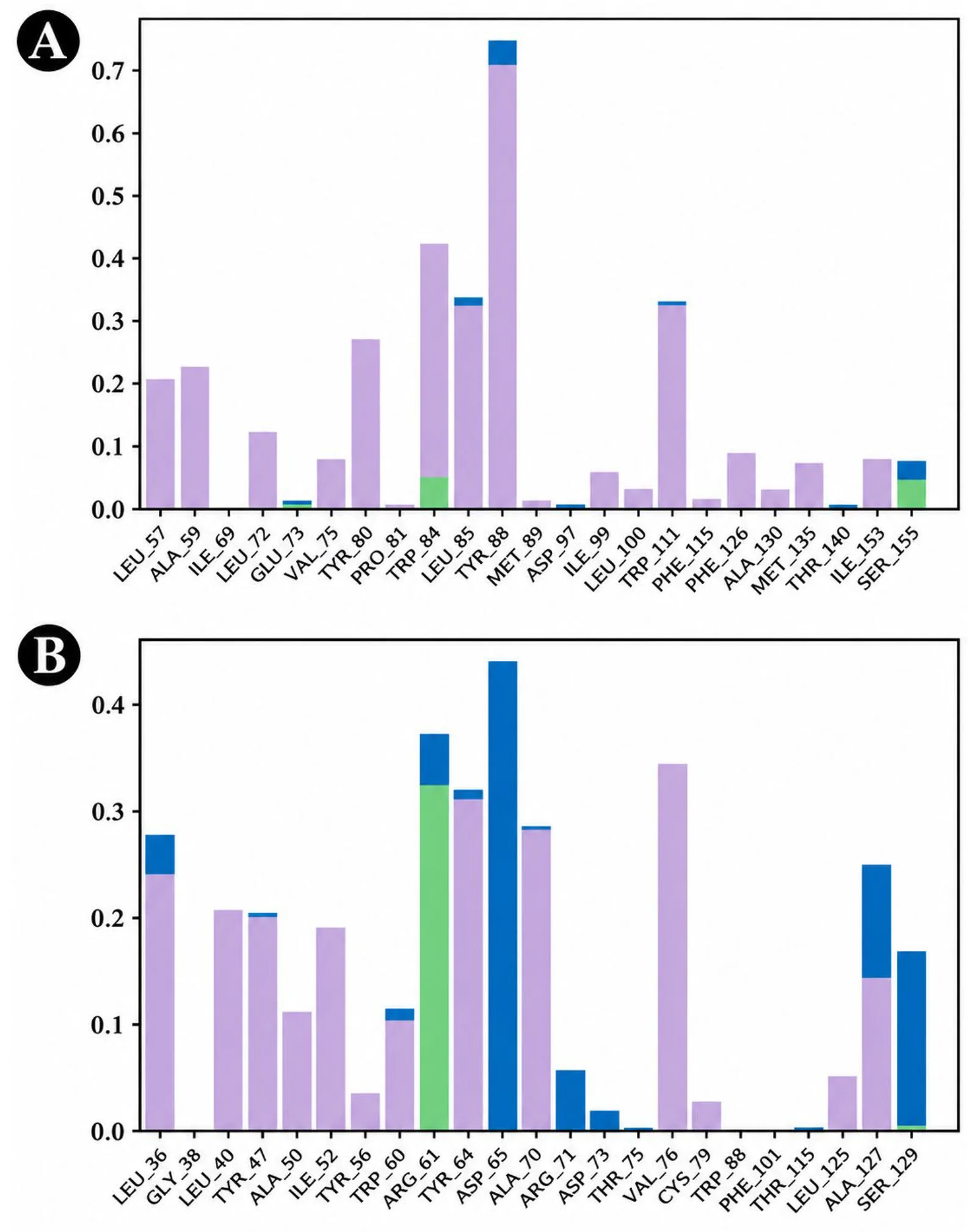
Interaction fraction analysis summarizing the key intermolecular contacts stabilizing (**A**) 2UV0–myricetin and (**B**) 3QP5–myricetin complexes. The plots highlight dominant residues contributing to ligand binding through persistent hydrogen bonding and hydrophobic interactions, which collectively support high-affinity complex formation. Different colors represent the corresponding interaction types identified during the trajectory analysis, including hydrogen-bonding and hydrophobic interactions.

Intermolecular interaction profiling (Figure 10A,B) revealed consistent contacts throughout the simulation. In the 3QP5–myricetin complex, key residues such as Trp84, Leu85, Tyr88, and Trp11 contributed predominantly through hydrophobic interactions that were maintained during the trajectory, ligand stability. In the 2UV0–myricetin complex, residues including Arg61, Tyr64, Ala70, and Val76 contributed to persistent ligand-protein interactions, with additional involvement of water-mediated interactions involving Asp65. The presence of sustained hydrophobic contacts and water bridges indicates maintenance of the protein–ligand interaction network under the simulated conditions.

#### 3.9.5. Principal Component Analysis (PCA)

**Figure 11:**
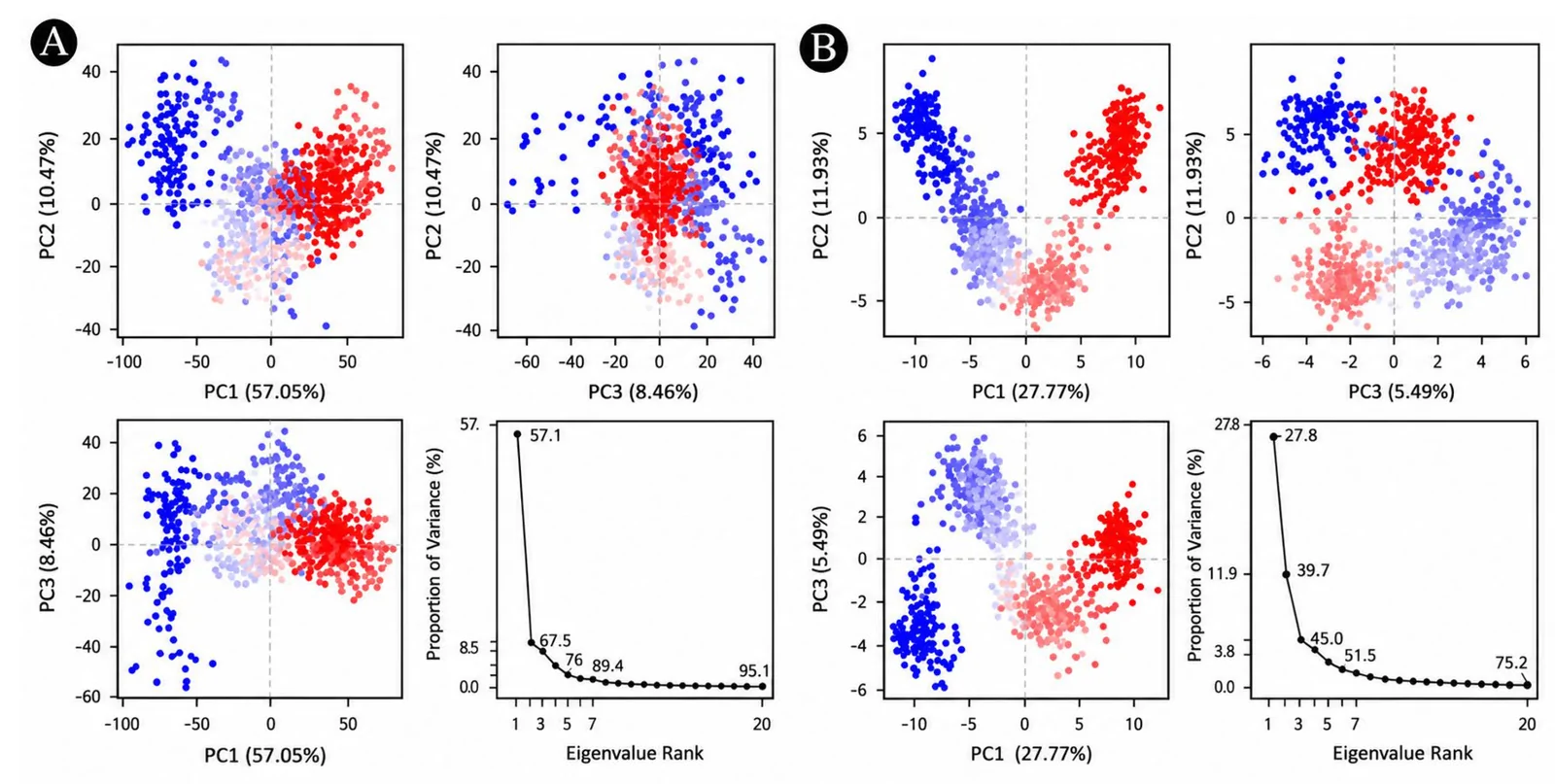
Principal component analysis (PCA) of the protein–myricetin complexes during the 100 ns molecular dynamics simulations. (**A**) PCA of the 2UV0–myricetin complex; (**B**) PCA of the 3QP5–myricetin complex. The PCA plots show the distribution and clustering of conformational states along the principal components, while the eigenvalue-rank plots illustrate the contribution of successive principal components to the overall variance. The different colors represent the corresponding conformational groups/states identified in the PCA analysis. The observed clustering indicates restricted collective motions and limited conformational variability during the simulations.

PCA was performed to examine large-scale conformational motions (Figure 11A,B). The 3QP5–myricetin complex exhibited compact clustering of conformations, indicating limited structural fluctuations. The dominant motion (PC1) corresponded to localized movements within the binding pocket. Similarly, the 2UV0–myricetin complex displayed tightly clustered conformations with minimal dispersion, suggesting conformational behavior conformational behaviour. The reduced conformational variability is consistent with the RMSD findings.

#### 3.9.6. Dynamic Cross-Correlation Matrix (DCCM) Analysis

**Figure 12:**
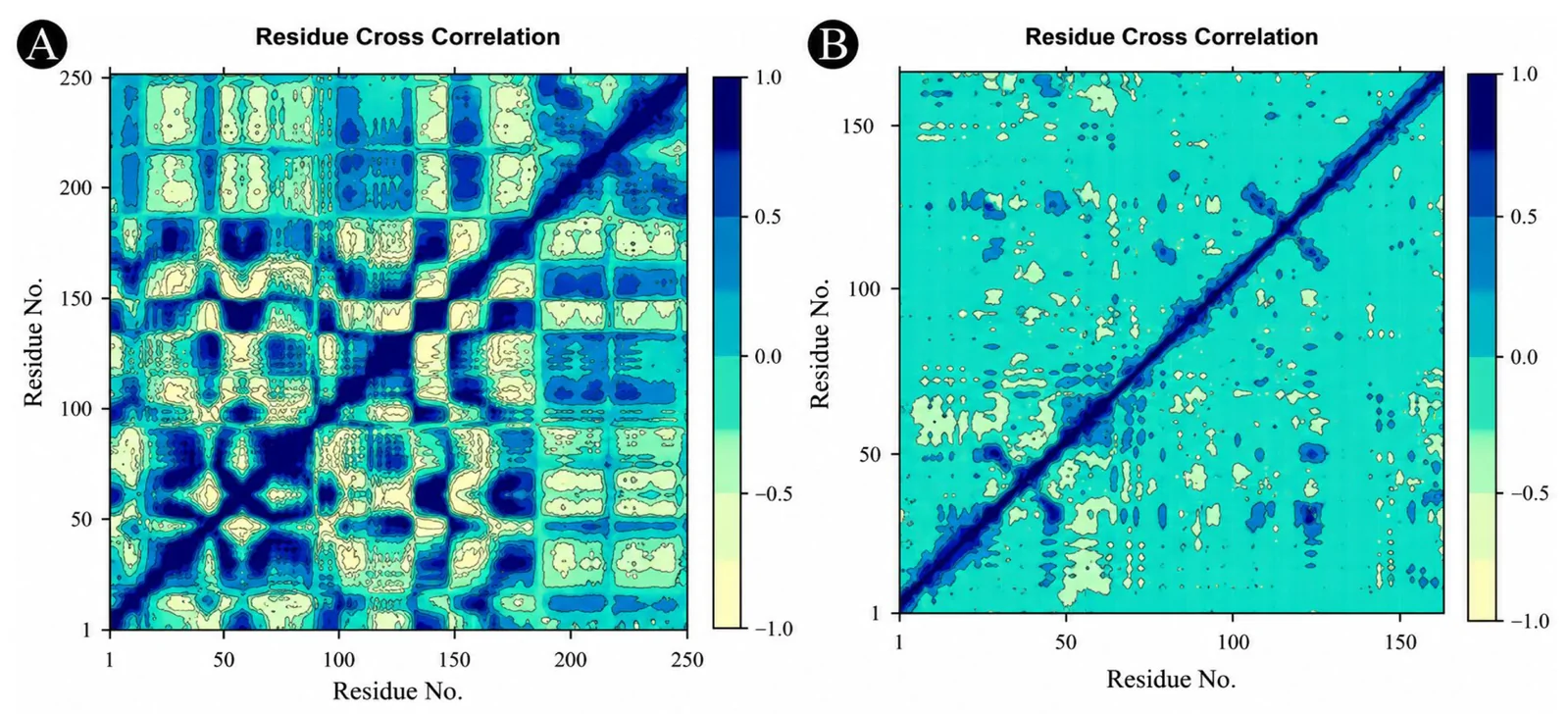
Dynamic cross-correlation matrix (DCCM) heatmaps illustrating correlated and anti-correlated residue motions in the (**A**) 2UV0 and (**B**) 3QP5 systems. The color scale represents the correlation coefficient, ranging from −1.0 to +1.0, with positive values indicating coordinated residue motions and negative values indicating anti-correlated motions. The predominantly positive correlations observed in both complexes indicate coordinated residue movements, while the relatively limited negative correlations suggest the absence of major opposing motions or domain separation during the simulations.

DCCM analysis revealed predominantly positive correlated (Figure 12A,B), particularly among residues surrounding the binding site, indicated coordinated residue movements. In the 3QP5–myricetin complex, correlated motions were observed between binding loops and adjacent α-helices, suggesting dynamic interactions within the binding region. Both complexes exhibited minimal anti-correlated motions, indicating the absence of major disruptions or domain separation during the simulation.

#### 3.9.7. Binding Free Energy Analysis (MM-GBSA)

The favorable negative binding energy estimates obtained by the Prime MM-GBSA calculations were obtained for both the protein–myricetin complexes at 100 and 0 ns. The 3QP5-myricetin complex showed a slightly better value for ΔG_bind of −57.874 kcal/mol compared to ΔG_bind of −54.841 for 2UV0-myricetin at 0 ns. The binding property of the two were reversed at 100 ns, where 2UV0-myricetin had a more negative ΔG_bind of −51.171 kcal/mol, while 3QP5-myricetin showed a less negative value of −46.256 kcal/mol. The MM-GBSA endpoint values are thus not consistent in terms of their energetic preference for one of the proteins along the trajectory. The change in ΔG_bind for 2UV0-myricetin between 0 and 100 ns was −3.670 kcal/mol. It was observed, however, that its van der Waals contribution improved from −32.204 to −33.536 kcal/mol and lipophilic contribution improved from −29.295 to −30.365 kcal/mol. Overall, these results show that the contribution of the dispersion and the hydrophobic interactions stayed high during the simulation, whereas the contribution of the hydrogen bonds stayed around 0.0. The small decrease in the total MM-GBSA estimate, therefore, could not be attributed to less hydrophobic interactions, but rather was linked mainly to the electrostatic and other components of the energy. The 3QP5-myricetin complex exhibited a greater change with ΔG_bind lowering by 11.618 kcal/mol from 0 to 100 ns. A major part of this change was due to the decrease in van der Waals contribution from −31.735 to −23.076 kcal/mol. The packing contribution also dropped from −3.412 to −0.661 kcal/mol. On the other hand, the lipophilic term turned more favorable and decreased from −29.937 to −32.530 kcal/mol, suggesting that myricetin had substantial hydrophobic interactions even after the van der Waals and packing contributions have weakened (Table 1). The hydrogen-bonding contribution was relatively small for both complexes, indicating that the energetic profiles calculated in the complexes were dominated by van der Waals and lipophilic interactions, rather than by the energy of direct hydrogen bonding. Overall, 2UV0-myricetin showed a lesser endpoint change over the time range of 0–100 ns than 3QP5-myricetin.

## 4. Discussion

The rapid increase in antimicrobial resistance (AMR), particularly among Gram-negative pathogens, necessitates the development of alternative therapeutic strategies that target bacterial virulence rather survival [1,34]. In this context, QS inhibition has emerged as a promising approach to suppress virulence while potentially reducing selective pressure for resistance development [35]. In the present study, myricetin demonstrated significant anti-virulence activity through both experimental and in silico analyses. The compound effectively modulated several QS-associated phenotypes, reduced biofilm formation and exhibited favourable computational interactions with key regulatory proteins.

Although myricetin showed moderate antibacterial activity, its primary significance lies in its effectiveness at sub-inhibitory concentrations, where it reduced several QS-associated and virulence-related phenotypes without exerting strong bactericidal effects. This is particularly important, as anti-QS strategies may reduce the likelihood of resistance development compared to conventional antibiotics [36,37]. An important consideration in interpreting anti-QS and anti-virulence effects is whether the tested concentrations affect bacterial growth. In the present study, growth assessment confirmed that myricetin at 1/8, 1/4 and 1/2 MIC did not significantly inhibit the growth of any of the tested bacterial strains. Therefore, the observed reductions in QS-regulated pigments, *P. aeruginosa* virulence factors and biofilm-associated phenotypes are unlikely to be explained simply by reduced bacterial growth. These findings support the interpretation that myricetin can modulate virulence-associated phenotypes at concentrations that do not significantly inhibit bacterial growth.

The observed reduction in violacein production in *C. violaceum* is consistent with modulation of AHL-associated QS-signaling [38]. Similarly, the suppression of prodigiosin in *S. marcescens* is consistent with broader QS-associated phenotypic modulation. These findings are consistent with previous reports describing flavonoids as modulators of LuxR-type receptors, suggesting that myricetin may competitively interfere with signal molecule binding or receptor activation [39,40]. Myricetin also reduced QS-controlled virulence factors in *P. aeruginosa*, including pyocyanin, pyoverdine, and rhamnolipids. These factors play important roles in oxidative stress induction, iron acquisition and biofilm maturation, respectively [16,41]. Their simultaneous reduction suggests that myricetin may influence QS-regulated processes such as those associated with the Las and Rhl systems [42]. Collectively, these findings support the potential of myricetin as a multi-target anti-virulence agent capable of modulating QS-associated pathogenic traits. Our findings in *P. aeruginosa* should be considered in the context of the recent study by Al Hashedi et al. [16], which reported that myricetin inhibited biofilm formation, QS-associated phenotypes and virulence-factor production in this organism. That study also reported reductions in pyocyanin, rhamnolipid and exopolysaccharide production and provided additional evidence through QS-related gene-expression analysis. Thus, the present *P. aeruginosa* findings are broadly consistent with the existing literature rather than representing a first demonstration of myricetin-mediated QS or inhibition of biofilm formation in this species. The contribution of the present study is instead the broader comparative evaluation across multiple bacterial species, including *S. aureus*, *E. coli*, *C. violaceum* and *S. marcescens*, together with the integration of multiple QS-associated and virulence-related phenotypes and structure-based computational analyses. This broader framework provides an opportunity to examine whether the phenotypic effects associated with myricetin extend beyond a single bacterial species.

The biofilm formation inhibition assay demonstrated that myricetin produced a concentration-dependent reduction in biofilm formation across both Gram-positive and Gram-negative bacteria. The reduction in biofilm biomass was associated with a significant decrease in EPS production. Since EPS forms the structural backbone of biofilms, its reduction may compromise biofilm integrity, potentially increasing the susceptibility of bacterial cells to environmental stress and antimicrobial agents [43]. Myricetin reduced EPS production across all tested strains, although the involvement of specific QS pathways was not established in this study. This dual effect on both signaling-associated and structural components supports the potential of myricetin to modulate biofilm formation. However, it should be noted that the crystal-violet assay used in the present study measures total attached biofilm biomass and does not independently distinguish viable from non-viable cells or demonstrate direct biofilm disruption or bacterial killing. Furthermore, myricetin was present during the biofilm-development period; therefore, the observed reduction in crystal-violet staining should be interpreted as inhibition or reduction in biofilm formation rather than treatment or eradication of an established mature biofilm. Assessment of viable biofilm cells by CFU enumeration and complementary microscopic or metabolic approaches would provide additional evidence regarding the effects of myricetin on established biofilms. Such analyses were beyond the scope of the present study.

To gain mechanistic insights at the molecular level, molecular docking was performed against key QS regulatory proteins. Myricetin exhibited favorable binding affinities, particularly with CviR (3QP5) and LasR (2UV0), which are central transcriptional regulators in QS systems [44]. Interaction analysis revealed hydrogen bonding, π–π stacking, and hydrophobic interactions, indicating stable ligand accommodation within the receptor pocket. These computational interactions suggest that myricetin may interfere with receptor function or autoinducer recognition however, direct biological target engagement was not experimentally confirmed in the present study. The ability of myricetin to adopt distinct binding conformations across different receptors further suggests structural flexibility and adaptability. These observations were further supported by molecular dynamics simulations. RMSD and RMSF analyses indicated that both protein–ligand complexes remained stable throughout the 100 ns simulation period, with minimal conformational deviations. Thus, the MD results provide additional computational support for the stability of the predicted complexes, but should not be interpreted as experimental confirmation of direct LasR or CviR binding. Reduced flexibility of residues within the binding pockets suggests that myricetin may influence dynamics. Secondary structure analysis showed preservation of the native protein fold, indicating structural compatibility of ligand binding [45,46].

Interaction profiling and PCA analyses provided supporting evidence of stable and coordinated binding. Persistent hydrophobic contacts and water-mediated interactions contributed to ligand stability, particularly in the 2UV0 complex. The compact clustering observed in PCA and the predominance of positive correlation in DCCM analysis indicate coordinated structural behavior without major destabilization [47]. These findings indicate that myricetin remained associated with the selected binding sites and that the corresponding protein–ligand complexes maintained relatively stable structural and interaction profiles during the simulations. However, because apo-protein simulations were not performed, these analyses do not establish ligand-induced stabilization or quantify changes relative to the unbound receptors. Binding free energy calculations further demonstrated favourable thermodynamic profiles for both complexes, with the 2UV0-myricetin complex showing slightly greater stability. The lower fluctuation and more consistent energy profile of this complex suggest enhanced electrostatic and van der Waals contributions, reinforcing its utility as a primary target [48]. Collectively, these computational findings are consistent with experimental observations, providing computationally supported mechanistic hypotheses for the observed QS-associated phenotypic and antibiofilm effects of myricetin.

This study demonstrates that myricetin has the potential to act as a multi-target anti-virulence agent, modulating QS-associated phenotypes, suppressing virulence factors and inhibiting biofilm formation under the tested conditions. Computationally predicted interactions with key regulatory proteins such as CviR and LasR suggest that myricetin may potentially interfere with bacterial communication processes. However, these predicted interactions require experimental validation. Importantly, its activity at sub-MIC levels highlights its potential for use in combination strategies, where it could enhance the efficacy of conventional antibiotics while reducing the likelihood of resistance development.

Despite these promising findings, several limitations should be acknowledged. First, the molecular docking and molecular dynamics analyses provide computational evidence for the predicted interaction and stability of myricetin with CviR and LasR, but direct biological target engagement was not experimentally validated in the present study. Experimental approaches such as competitive ligand-binding assays, thermal shift assays, or site-directed mutational studies would be required to confirm these predicted interactions. Second, although QS-associated phenotypic, anti-virulence, and biofilm formation-inhibitory effects were demonstrated under in vitro conditions, the biological efficacy of myricetin has not yet been evaluated in relevant in vivo infection models. In addition, pharmacokinetic, bioavailability, toxicity and dose–response studies are required to determine its suitability for therapeutic development. Therefore, the present findings should be considered preliminary evidence supporting the anti-virulence potential of myricetin rather than confirmation of its therapeutic efficacy. Future studies integrating experimental target validation and in vivo evaluation will be necessary to establish its clinical relevance. An additional limitation is that the crystal violet assay used in this study measures total attached biofilm biomass and does not distinguish between viable and non-viable bacterial cells within the biofilm. Therefore, the observed reduction in crystal violet staining should be interpreted as a reduction in total biofilm biomass rather than direct evidence of reduced bacterial viability. Future studies using viability-based assays would be useful to further determine the effect of myricetin on the viability of biofilm-associated cells.

## 5. Conclusions

The present study provides experimental evidence that myricetin exhibits anti-virulence potential, with the ability to modulate QS-associated phenotypes and inhibit biofilm formation across multiple pathogenic bacteria under the tested conditions. At sub-inhibitory concentrations, myricetin significantly reduced QS-associated pigments, virulence determinants, EPS production and biofilm formation under the tested experimental conditions. These findings represent experimentally observed phenotypic effects and should not be interpreted as definitive evidence of direct QS inhibition. Complementary computational analyses predicted favourable interactions of myricetin with selected QS and virulence-associated proteins, particularly CviR and LasR. These computational results provide mechanistic hypotheses regarding potential myricetin–protein interactions rather than experimental confirmation of direct receptor binding or target engagement. The present study was limited to in vitro investigations and direct QS signaling measurements, transcriptional analyses and experimental target-validation studies were not performed. Therefore, the therapeutic efficacy, pharmacokinetic properties, safety and in vivo activity of myricetin remain to be established. Further studies involving direct QS-specific assays, biological target validation and appropriate in vivo infection models are necessary to determine whether the observed anti-virulence and antibiofilm effects can be translated into therapeutic applications. Overall, the present findings extend previous reports of myricetin-associated antibiofilm, QS-related and anti-virulence effects, particularly those described in *P. aeruginosa*, by evaluating a broader panel of bacterial species and integrating multiple phenotypic endpoints with molecular docking and molecular dynamics analyses. The experimental findings support the potential of myricetin as a biofilm-formation and virulence-modulating compound, while the computational analyses provide testable hypotheses regarding its potential interactions with bacterial regulatory proteins.

## Figures and Tables

**Table 1:** MM-GBSA binding free energy profile of the myricetin–protein complexes at 0 and 100 ns. Values are reported in kcal/mol.

Complexes	Time (ns)	ΔG_Bind	ΔG_vdW	ΔG_Lipo	ΔG_Hbond	ΔG_Packing
2UV0-Myricetin	0	−54.841	−32.204	−29.295	−0.039	−0.005
2UV0-Myricetin	100	−51.171	−33.536	−30.365	−0.001	−0.668
3QP5-Myricetin	0	−57.874	−31.735	−29.937	−0.237	−3.412
3QP5-Myricetin	100	−46.256	−23.076	−32.530	−0.004	−0.661

## Data Availability

The data supporting the findings of this study are presented within the manuscript, including the experimental results, summary statistics, and computational findings. The complete underlying raw experimental measurements and detailed computational output files, including individual replicate-level measurements, docking configuration files, trajectory-level analyses, and MM-GBSA output files, are not currently deposited in a public repository. Additional information regarding the experimental and computational procedures is provided in Section 2 (Materials and Methods).
